# Structural mapping of antibody landscapes to human betacoronavirus spike proteins

**DOI:** 10.1126/sciadv.abn2911

**Published:** 2022-05-04

**Authors:** Sandhya Bangaru, Aleksandar Antanasijevic, Nurgun Kose, Leigh M. Sewall, Abigail M. Jackson, Naveenchandra Suryadevara, Xiaoyan Zhan, Jonathan L. Torres, Jeffrey Copps, Alba Torrents de la Peña, James E. Crowe, Andrew B. Ward

**Affiliations:** 1 Department of Integrative Structural and Computational Biology, The Scripps Research Institute, La Jolla, CA 92037, USA.; 2 The Vanderbilt Vaccine Center, Vanderbilt University Medical Center, Nashville, TN 37232, USA.; 3 Departments of Pathology, Microbiology, and Immunology, Vanderbilt University, Nashville, TN 37232, USA.; 4 Department of Pediatrics, Vanderbilt University, Nashville, TN 37232, USA.

## Abstract

Preexisting immunity against seasonal coronaviruses (CoVs) represents an important variable in predicting antibody responses and disease severity to severe acute respiratory syndrome CoV-2 (SARS-CoV-2) infections. We used electron microscopy–based polyclonal epitope mapping (EMPEM) to characterize the antibody specificities against β-CoV spike proteins in prepandemic (PP) sera or SARS-CoV-2 convalescent (SC) sera. We observed that most PP sera had antibodies specific to seasonal human CoVs (HCoVs) OC43 and HKU1 spike proteins while the SC sera showed reactivity across all human β-CoVs. Detailed molecular mapping of spike-antibody complexes revealed epitopes that were differentially targeted by preexisting antibodies and SC serum antibodies. Our studies provide an antigenic landscape to β-HCoV spikes in the general population serving as a basis for cross-reactive epitope analyses in SARS-CoV-2–infected individuals.

## Teaser

Structural analysis of human polyclonal serum antibodies reveals diverse responses to seasonal β-CoV spikes.

## INTRODUCTION

Four human coronaviruses (HCoVs) of genus α (HCoV-229E and HCoV-NL63) or β (HCoV-OC43 and HCoV-HKU1) are endemic in the human population contributing up to a third of the common cold infections (*1*, *2*). While the infection rate and prevalence of these HCoVs vary on the basis of the region, primary infections occur early in life with a majority of the population infected before 15 years of age (*2*–*5*). Most individuals possess antibodies to HCoVs targeting the trimeric spike glycoprotein and the nucleocapsid protein (N) although antibodies wane over time permitting reinfection even within a year (*6*–*9*). In addition to HCoVs OC43 and HKU1, the β-CoV genus also contains three highly pathogenic CoVs associated with human disease: Middle East respiratory syndrome (MERS) CoV, severe acute respiratory syndrome (SARS) CoV, and the novel SARS-CoV-2, the causative agent of the ongoing coronavirus disease 2019 (COVID-19) pandemic (*10*, *11*).

The spike protein is an important determinant of host range and cell tropism because it mediates virus attachment and entry into the host cells, making it a major target for neutralizing antibodies and a key component for vaccine development (*12*–*16*). While the SARS-CoV-2 spike shares high structure and sequence homology with the SARS (69.2%) spike, it is less conserved across other β-CoVs, with as little as 27.2% sequence homology between SARS-CoV-2 and OC43 (*17*). Despite the low sequence conservation, preexisting immunity against seasonal CoV spike proteins has been associated with COVID-19 disease outcome as a consequence of either back-boost or induction of cross-reactive antibodies following SARS-CoV-2 infection (*8*, *9*, *18*–*22*). Of interest, SARS-CoV-2 convalescent (SC) donors with high SARS-CoV-2 antibody titers also possessed increased levels of antibodies against β-HCoVs (*8*, *23*). It is not clear if infection triggers a recall of preexisting HCoV-specific antibodies or preferentially elicits cross-reactive β-CoV antibodies targeting conserved epitopes. Here, we elucidate the β-HCoV spike epitopes targeted by preexisting serum antibodies and compare them to those elicited following SARS-CoV-2 infection using electron microscopy–based polyclonal epitope mapping (EMPEM) methodology (*24*, *25*).

## RESULTS AND DISCUSSION

### Serum reactivity to β-CoV spikes

**Fig. 1.**
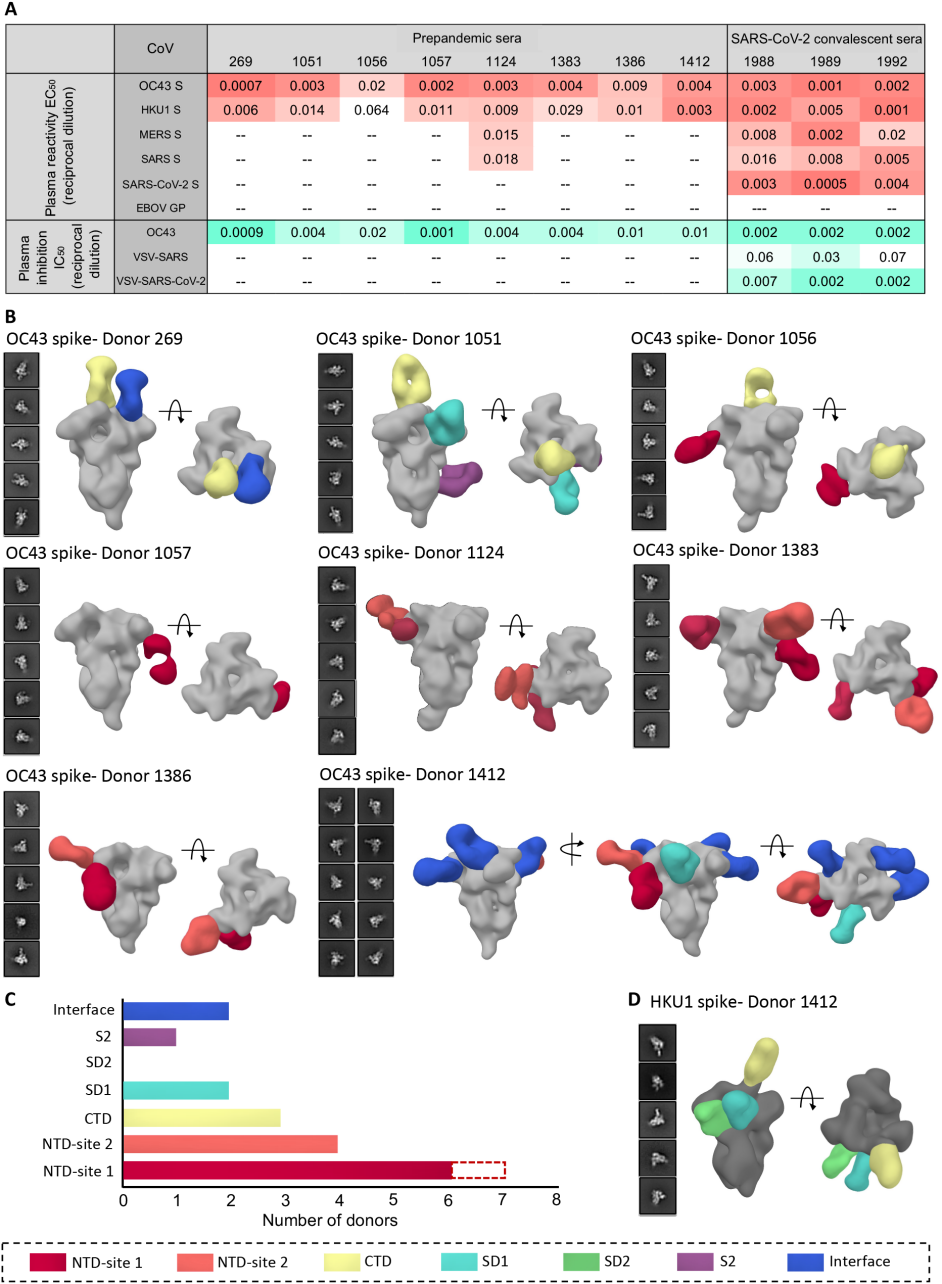
Human serum reactivity to β-CoV spikes. (**A**) ELISA EC_50_ binding titers to OC43, HKU1, MERS, SARS, and SARS-CoV-2 spikes and median inhibitory concentration (IC_50_) neutralization titers against OC43 virus and vesicular stomatitis virus (VSV)–pseudotyped SARS or SARS-CoV-2 virus for PP sera from eight healthy donors and SC sera from three SARS-CoV-2 donors. Ebola virus glycoprotein (EBOV GP) was used as a negative control for detecting nonspecific serum binding. Serum EC_50_ or IC_50_ titers are color-coded in gradients of orange or aquamarine, respectively. (**B**) Representative two-dimensional (2D) classes and side and top views of composite figures from ns-EMPEM analysis of polyclonal Fabs from eight PP sera with the OC43 spike. (**C**) Bar graph summary of OC43 spike epitopes targeted by PP donor sera. Antibodies to NTD-site 1 were observed in 2D class averages for donor 269 but did not reconstruct in 3D, as indicated by dotted lines. (**D**) Composite figures from ns-EMPEM analysis of polyclonal Fabs from donor 1412 with the HKU1 spike. The Fabs in (B) and (D) are color-coded on the basis of their epitope specificities as indicated at the bottom. OC43 or HKU1 spikes in (B) and (D) are represented in light gray or dark gray, respectively.

Soluble ectodomains of spike proteins for β-CoVs, HKU1, OC43, SARS, MERS, and SARS-CoV-2 (four stabilized constructs were used for SARS-CoV-2) were generated and characterized by negative stain electron microscopy (ns-EM) and shown to be homogeneous in their prefusion conformation (fig. S1A). To determine the baseline serum antibody titers to β-CoV spikes in the general population, either sera or plasma (on the basis of availability) collected before the COVID-19 pandemic from eight healthy donors with unknown HCoV infection history was screened for spike antibodies by enzyme-linked immunosorbent assay (ELISA). All eight donors exhibited reactivity to the OC43 spike, with median effective concentration (EC_50_) serum dilution values ranging from 0.0007 to 0.02, while HKU1 antibody titers were lower in general (serum dilution EC_50_ of 0.001 to 0.06) (Fig. 1A and fig. S1B). This finding is consistent with OC43 being the most commonly encountered HCoV globally while HKU1 is less prevalent (*4*, *5*). Reactivity against SARS-CoV-2 spike was not detected in any of the prepandemic (PP) sera, and only one of eight donors (D1124) exhibited low-level reactivity against SARS and MERS spikes. For comparison, we then assessed β-CoV spike reactivity in three SC serum samples (~day 56 after infection), all of which exhibited high antibody titers to SARS-CoV-2 spike. Notably, the three donors also showed reactivity against other β-CoV spikes including SARS and MERS (Fig. 1A and fig. S1B). The overall difference in antibody titers to these pathogenic CoVs in SC sera as compared to PP sera indicate that SARS-CoV-2 infection can elicit some level of cross-reactive responses against the β-CoV spikes. While OC43 reactivity was high in both PP and SC sera, HKU1 spike antibody titers appeared enhanced in SC sera (Fig. 1A and fig. S1B). To investigate whether serum antibody reactivity translated to inhibitory activity, we performed neutralization assays with both PP and SC sera against the OC43 virus and vesicular stomatitis virus (VSV)–pseudotyped SARS and SARS-CoV-2 viruses. Overall, the serum inhibitory titers against the OC43 virus correlated well with their binding titers (Fig. 1A and fig. S1C). While none of the PP sera neutralized SARS or SARS-CoV-2 virus, the SC sera exhibited neutralizing activity [serum dilution median inhibitory concentration (IC_50_) of 0.002 to 0.007] against the SARS-CoV-2 virus and some weak activity against the SARS virus (Fig. 1A and fig. S1C).

### Ns-EMPEM analysis of preexisting serum antibodies to β-CoV spikes

We next used ns-EMPEM to determine the epitope specificities of spike antibodies in the PP sera. Structural analysis of polyclonal Fabs complexed with spike proteins from OC43, HKU1, SARS, or MERS revealed OC43-reactive antibodies in all eight donors and HKU1-reactive antibodies in one donor (Fig. 1, B to D). We did not detect antibodies to either SARS or MERS spikes. Published cryo-EM structures of β-CoV spikes show the cleavable S1 and S2 subunits comprising an N-terminal domain (NTD), a C-terminal domain (CTD), subdomains 1 and 2 (SD1 and SD2), the fusion peptide (FP), and heptad repeats 1 and 2 (HR1 and HR2) (*26*–*29*). OC43 NTD-reactive Fabs were seen in seven donors targeting either the 9-O-acetylated sialic acid receptor binding site (RBS) defined by loop residues 27 to 32, 80 to 86, 90, and 95 (NTD-site 1) or a site adjacent to the RBS encompassing residues from loops 112 to119, 176 to 186, and 254 to 261 (NTD-site 2; Fig. 1, B and C). The prevalence of NTD-site 1 Fabs that can sterically block receptor engagement correlated well with the OC43 inhibitory titers observed in PP sera across all donors. While neither the CTD nor the SD1 of OC43 spike is associated with any known function, antibodies to CTD were seen in at least three donors, to SD1 in two donors, and to S1 interprotomeric interfaces in two donors. A single S2-reactive antibody from donor 1051 displayed a broad footprint with potential interactions with residues 800 to 807, 1013 to 1031, and 1062 to 1068 (fig. S1D). Of interest, donor 1412 with a relatively low OC43 neutralization titer displayed the greatest diversity of Fab specificities, targeting six distinct S1 epitopes including the interprotomeric interfaces. This individual was also the only donor with detectable Fab responses to the HKU1 spike targeting the CTD, the SD1, and the SD2 (Fig. 1D).

### Cryo-EMPEM analysis of PP serum antibodies from healthy donors to OC43 spike

**Fig. 2.**
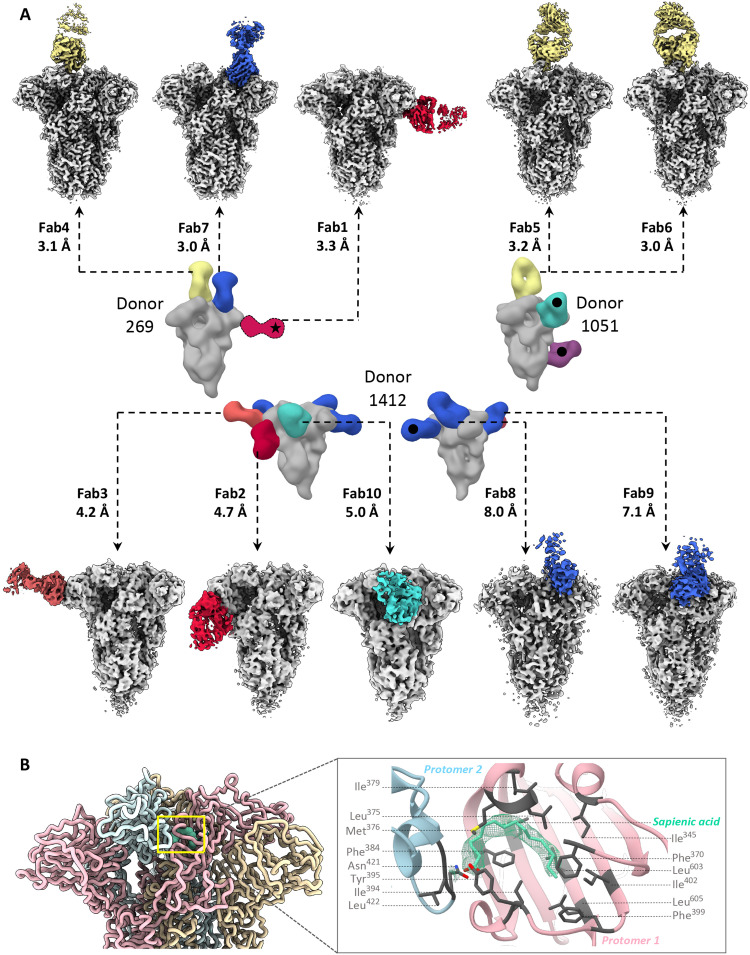
Cryo-EMPEM analysis of OC43 spike-polyclonal Fab complexes. (**A**) High-resolution cryo-EMPEM reconstructions of OC43 spike complexed with polyclonal Fabs derived from PP sera from donors 269 (top left), 1051 (top right), or 1412 (bottom); the representative composite figures from ns-EMPEM from these donors are shown in the middle. Each map depicts a structurally unique polyclonal antibody class reconstructed at the indicated resolution with the Fabs colored according to the scheme used in Fig. 1. OC43 spike is represented in light gray. Fabs marked with a black dot were observed by ns-EMPEM but were not detected by cryo-EMPEM. Fab class from donor 269 marked with a star was resolved by cryo-EMPEM but not by ns-EMPEM. (**B**) Sapienic acid (aquamarine) binding within a hydrophobic pocket in the CTD-CTD interprotomeric interface. Protomers are colored in light pink, blue, or wheat and the interacting residues are shown in gray.

Samples from three donors (269, 1051, and 1412) were chosen for high-resolution cryo-EMPEM studies with OC43 spike as they represented individuals with antibodies against all the unique epitopes observed (table S1). We reconstructed 10 high-resolution maps of unique spike-Fab complexes (Fig. 2A, figs. S2 to S5, and table S2). High-resolution analysis of immune complexes from donor 269 revealed Fabs bound to the CTD, CTD-NTD interface, and NTD-site 1; NTD-site 1 Fab was not reconstructed during the ns-EMPEM studies. For donor 1051, cryo-EM analysis enabled differentiation of polyclonal Fabs targeting the CTD that were originally observed as a single species by ns-EM. We were unable to obtain reconstructions of either the SD1 or S2 antibody despite multiple attempts at focused classification, likely owing to low Fab abundance or dissociation of the complex during the cryogenic sample preparation process. For donor 1412, we reconstructed five of the six specificities seen in ns-EMPEM, targeting NTD-site 1, NTD-site 2, SD, and interprotomeric S1 interfaces.

In all reconstructed maps, we observed an additional nonspike density buried within a hydrophobic pocket in the CTD; the location and size resembling linoleic acid in SARS-CoV-2 spike (*30*, *31*). The *M*_w_ of 254 g/mol obtained by mass spectrometry analysis of the OC43 spike and the corresponding density in the OC43 map are however consistent with sapienic acid (6Z-hexadecenoic acid; Fig. 2B and fig. S5A). The aliphatic chain of sapienic acid improves the hydrophobic packing at the CTD-CTD interface of two adjacent protomers, while the carboxyl group forms hydrogen bonds with the side chain of Tyr^395^ in the CTD of one protomer and the main chains of residues Leu^422^ and/or Gly^423^ within the CTD of the other protomer (Fig. 2B). These contacts likely help stabilize the closed conformation of OC43 spike.

**Fig. 3.**
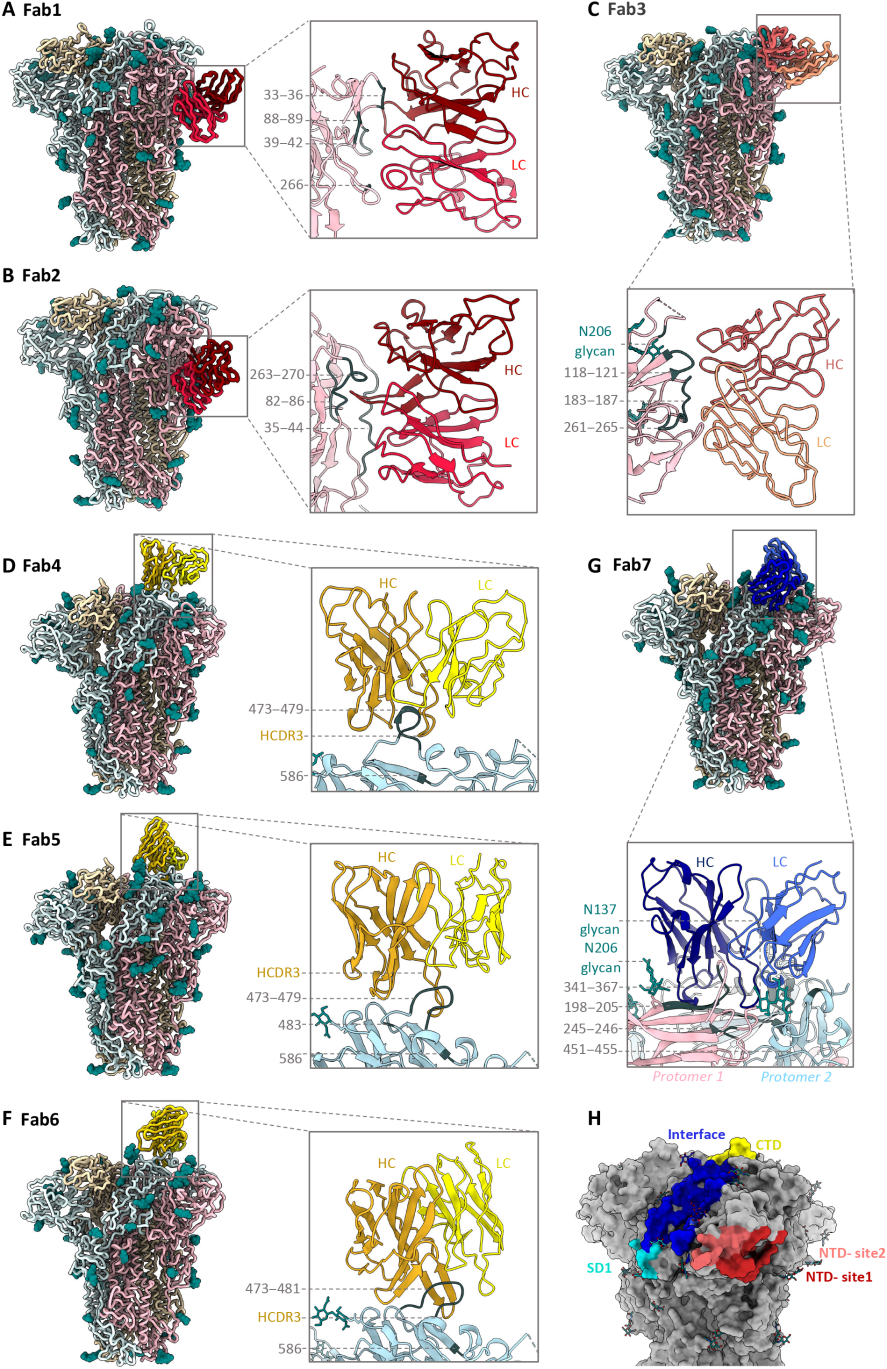
Cryo-EM structures of polyclonal Fabs targeting the OC43 spike. (**A** to **G**) Tube or ribbon representation of atomic models of OC43 spike-Fab complexes along with zoomed-in views of epitope-paratope interactions. (A and B) Fab1 and Fab2 (red) target the NTD-site1 or RBS; (C) Fab3 (orange) targets NTD-site2 adjacent to RBS; (D to F) Fab4, Fab5, and Fab6 (yellow) target the CTD; and (G) Fab7 (blue) targets the NTD-CTD interface. The spike protomers are shown in light blue, light pink, or wheat (ribbon representation) with glycans in teal (sphere atom representation) and primary epitope contacts in gray. Detailed contact residues along with corresponding EM densities are shown in figs. S6 and S7. (**H**) Surface representation of OC43 spike (gray) showing collective epitopes of Fab1 to Fab10 colored on the basis of their binding site using the color scheme from Fig. 1.

Atomic models of spike-Fab complexes were relaxed in 7 of 10 maps with resolutions ≤4.8 Å (Fig. 3, A to G, and figs. S6 and S7). Fabs were represented as poly-alanine pseudo-models. Both Fabs to immunodominant NTD-site 1 (Fab1-spike at 3.3 Å and Fab2-spike at 4.7 Å) approach the RBS at different angles with dissimilar engagement of antibody heavy- and light-chain complementarity-determining regions (HCDR and LCDR; Fig. 3, A and B, and fig. S6, A and B). While Fab1 made spike contacts at residues 33 to 36 (using HCDR2), 39 to 42 (HCDR3), 88 to 89 (LCDR3), and 264 to 267 (LCDR1), Fab2 approaches at a much steeper angle by inserting its HCDR3 into the NTD pocket encompassing loops 82 to 86, 35 to 43, and 263 to 270 along with other LCDR1 and LCDR2 contacts at residues 40 to 44. Fab3 (4.2 Å), targeting the second prevalent site, NTD-site 2, binds adjacent to RBS with main contacts at residues 118 to 121 (LCDR3) and interacting with loops 183 to 187 (HCDR3) and 261 to 265 (LCDR1) (Fig. 3C and fig. S6C). While antibodies to NTD-site 1 directly overlap with the RBS, NTD-site 2 antibodies could potentially block receptor binding by steric hindrance. Collectively, cryo-EMPEM analysis of Fabs to NTD reveals structural features of these immunodominant epitopes associated with antiviral activity against OC43.

High-resolution reconstructions of three CTD Fab-spike complexes (Fab4 at 3.1 Å, Fab5 at 3.2 Å, and Fab6 at 3.0 Å) reveal an almost identical epitope featuring a single CTD loop 472 to 483 (Fig. 3, D to F, and fig. S6, D to F). Fab4 surrounds the loop with HCDR1, HCDR2, HCDR3, and LCDR3 making contacts with residues 473 to 479, and Trp^586^. Whereas Fab4 binding did not induce any conformational changes in the loop residues 472 to 483 in comparison to the published OC43 apo-spike structure [Protein Data Bank (PDB) no. 6OHW; (*29*)], both Fab5 and Fab6 stabilize the loop in a different conformation (Fig. 3, D to F, and fig. S6, D to F). While Fab5 uses HCDR2, HCDR3, LCDR1, and LCDR3 to interact with loop residues 474 to 477 and 483 with potential HCDR3 contact at Trp^586^, Fab6 binds in a similar manner with the main distinguishing features being HCDR2 interaction with Thr^481^ instead of His^483^, additional HCDR1 contact with Val^479^, and the displacement of glycan at position Asn^449^ by the longer Fab6 LCDR1 (Fig. 3, E and F, and fig. S6, E and F). Overall, structural analysis of antibodies to CTD reveals the loop 472 to 483 as the major antigenic element that is generally sandwiched between multiple CDRs with Trp^586^ stabilizing the interaction.

Among the three interprotomeric antibodies reconstructed, Fab7 (3 Å) and Fab8 (8 Å) bind two structural domains (CTD and NTD) while Fab9 (7.1 Å) extends across three domains (NTD, CTD, and SD1; Fig. 3G and fig. S7, A to C). Fab7 interacts extensively with both NTD (residues 140, 169, 198, 200 to 205, and 245 to 246) and CTD (residues 341 to 367, 451 to 455, and 469) using all CDRs, although the main interactions are facilitated by HCDR3. The antibody makes glycan contacts at position Asn^206^ while shifting the Asn^137^ glycan from its original position to accommodate LCDR1 (Fig. 3G and fig. S7A). Fab8 epitope, composed of NTD loop 196 to 208 and CTD loop 339 to 352, is bordered by four glycans with potential contacts with glycans at positions Asn^206^ and Asn^449^ and the Fab9 interaction is driven by NTD loops 204 to 210 and 149 to 159 on the first protomer with potential Asn^206^ glycan contact and by two CTD loops 337 to 341 and 366 to 367 and two SD1 loops 672 to 676 and 622 to 624 on the adjacent protomer (fig. S7, B and C). Notably, the Asn^675^ glycan is buried in the Fab9 spike interface making contact with the antibody (fig. S7C). Last, Fab10 (5 Å) targets the SD1 with primary interactions with Asp^624^, Glu^646^, Arg^676^, and glycans at Asn^648^ and Asn^678^ (fig. S7D). Fab9 and Fab10 both make extensive contacts to the Asn^675^ glycan, which represents an important immunogenic determinant within the SD1 epitope. An epitope summary of commonly elicited β-CoV spike antibodies in healthy human serum is shown in Fig. 3H and CDR lengths for Fab1 to Fab7 determined by structural homology are summarized in table S3.

### Epitope mapping of polyclonal antibodies to β-CoV spikes in SC sera

**Fig. 4.**
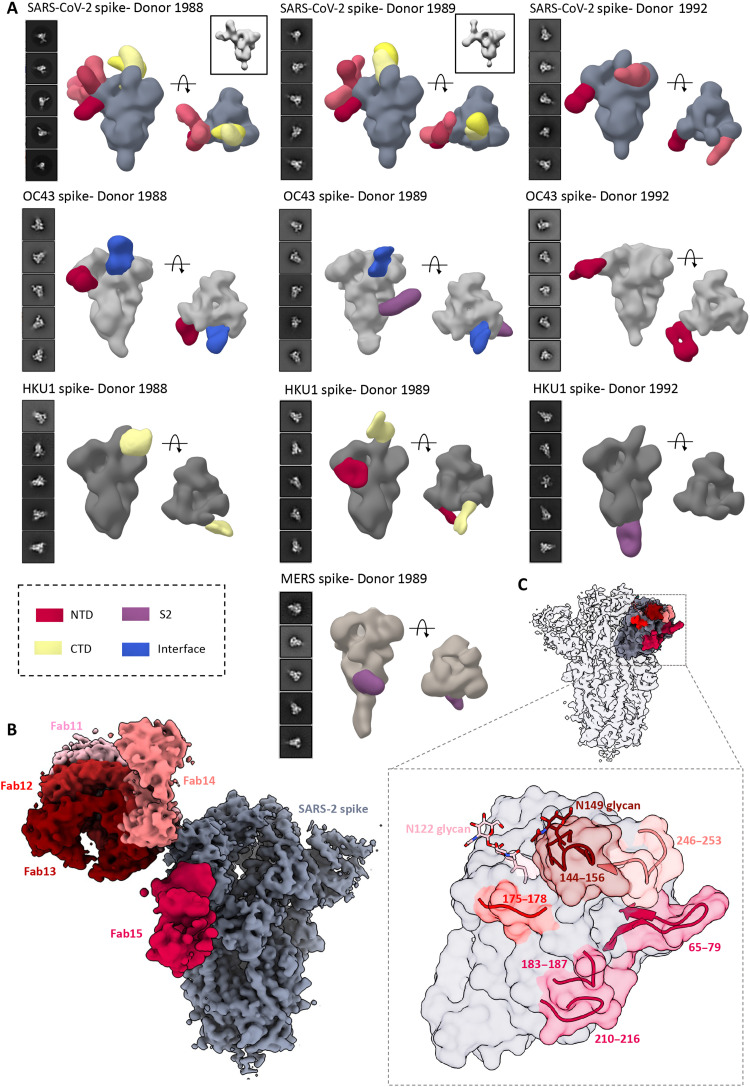
ns- and cryo-EMPEM analysis of polyclonal Fabs from SC donor sera. (**A**) Representative 2D classes and side and top views of composite figures from ns-EMPEM analysis of polyclonal Fabs from three SC donors complexed with β-CoV spikes. The donor numbers along with the corresponding CoV spikes are indicated above each panel in (A). The Fabs are color-coded on the basis of their epitope specificities as indicated at the bottom left. SARS-CoV-2, OC43, HKU1, and MERS spikes are represented in slate gray, light gray, dark gray, and beige, respectively. Three-dimensional reconstructions displaying potential self-reactive antibodies are shown in gray on the top right corners for both donors 1988 and donor 1999 in complex with SARS-CoV-2 spike. (**B**) Composite figure showing five unique antibody classes, Fab11 to Fab15 colored in shades of red, to SARS-CoV-2 spike NTD reconstructed using cryo-EMPEM analysis of polyclonal Fabs from donors 1988 and 1989 complexed with SARS-CoV-2–stabilized spikes. (**C**) Surface representation of SARS-CoV-2 spike showing epitopes of Fabs 11 to 15 from (B) on a single NTD (slate gray) with a zoomed-in view displaying the loop residues comprising each epitope. Loop 144 to 156 with the N149 glycan forms an immunodominant element commonly targeted by Fabs 11 to 14. The sub-epitope colors correspond to each Fab shown in (B).

Next, we sought to investigate the nature of spike antibodies in serum following SARS-CoV-2 infection. SC sera from three donors were screened for antibodies to SARS-CoV-2 spike by ns-EMPEM. Analysis of EM data [two-dimensional (2D) and 3D] revealed both NTD and RBD (or CTD) antibodies, although the latter were relatively fewer in number and more difficult to reconstruct owing to the flexible RBD (Fig. 4A). While RBD antibodies have been well documented to provide protection against SARS-CoV-2 infection, NTD responses are being recognized as an important component of the neutralizing response to SARS-CoV-2, particularly those targeting the supersite consisting of residues 14 to 20, 140 to 158, and 245 to 264 (*32*–*37*). Collectively, these donors possessed several polyclonal antibodies targeting this supersite along with antibodies to other previously described sites (*32*). We observed antibody pairs in both donors 1988 and 1989 with overlapping EM densities that could indicate a complex epitope composed of antibody and NTD (Fig. 4A). It is unclear why these antibodies are triggered in SARS-CoV-2 donors, and the implications of this finding for understanding COVID pathology need further investigation.

To obtain detailed molecular information on immunodominant epitopes within the SARS-CoV-2 spike, we subjected polyclonal samples from two SC donors (pooled Fabs from donors 1988 and 1989) to cryo-EMPEM analysis with SARS-CoV-2 spike. The analysis yielded five maps featuring NTD antibodies (Fab11 to Fab15; Fig. 4, B and C, and figs. S8 and S9, and table S4). Fabs 11 to 14 were reconstructed at resolutions 3.9, 4.2, 4.3, or 4.4 Å, respectively, and were all immunofocused onto the NTD loop 145 to 155 with the Asn^149^ glycan present at the core of each interaction (Fig. 4, B and C). Fab11 with its tilted angle of approach also made contacts with the Asn^122^ glycan while Fab12 and Fab13 appeared to make some additional contacts with adjacent loops 176 to 181 and 247 to 252. Although Fab14 also binds to loop 145 to 155, its distinctly different angle of approach also allows extensive contacts with loop 246 to 253, similar to supersite antibodies (*32*). In contrast, Fab15 interacts with loops 65 to 79, 183 to 187, and 210 to 217, an antigenic site similar to that recognized by the antibody S2M24 (*32*).

Our ELISA data demonstrated that there is an increase in antibody binding titers to non–SARS-CoV-2 β-CoV spikes following an infection with SARS-CoV-2 virus (“convalescent donors;” Fig. 1A). This finding indicates either a back-boost of preexisting responses or elicitation of cross-reactive antibodies to conserved epitopes. Structural mapping of SARS-CoV-2 spike residues that are either identical to or have a conserved substitution in at least three of the four other β-CoVs, OC43, HKU1, SARS, and MERS, revealed several conserved patches in the S2 subunit that could potentially elicit cross-reactive responses (fig. S10A). Several recent studies have found S2 as a target for cross-reactivity across β-CoVs (*8*, *9*, *18*, *19*, *22*). Two individual studies also revealed SARS-CoV-2 spike residues in and around 560 to 572, 819 to 824, and 1150 to 1156 and their homologous regions on other HCoV spikes as being recognized with higher frequency in patients with COVID-19 as compared to pre-COVID controls (fig. S10A) (*18*, *20*). To determine whether these epitopes are targeted following SARS-CoV-2 infection, we performed ns-EMPEM on SC sera with OC43, HKU1, SARS, and MERS. As with PP sera, the SC sera had antibodies to the OC43 spike protein; antibodies to NTD-site 1 were seen in two donors, antibodies to interface were seen in two donors, and an S2 antibody was observed in one donor (Donor 1989; Fig. 4A). While we are uncertain whether the S2 antibody was induced by SARS-CoV-2 infection, the antibody appears to target the helix 1014 to 1030 that is highly conserved across the β-CoV spikes (fig. S10B). Notably, donors who possessed high levels of OC43 antibodies also had some SARS-CoV-2–reactive antibodies prepandemic that did not correlate with protection against SARS-CoV-2 (*9*). When complexed with the HKU1 spike, we were able to detect antibodies in all three SC serum samples, which was higher than seen in PP sera (3D reconstructions were possible only for one of the eight PP donor sera), suggesting an increase in HKU1 antibody titers following SARS-CoV-2 infection (Fig. 4A). Of interest, Song *et al.* (*8*) observed higher HKU1 spike antibody titers in post-COVID sera compared to PP sera, whereas titers remained comparable for other HCoV spikes. Whereas donors 1988 and 1989 had antibodies to the HKU1 CTD and/or the NTD, donor 1992 sera contained an S2 antibody binding to the base of the trimer. The epitope is analogous to that of the β-CoV cross-reactive spike monoclonal antibody (mAb) CC40.8 isolated from a COVID donor (fig. S10C); CC40.8 binds strongly to SARS-CoV-2 and HKU1 spikes while also exhibiting some reactivity to SARS and OC43 spikes (*8*). We also reconstructed a MERS spike antibody in donor 1989 that partly overlaps with the known MERS mAb G4 targeting the S2 connector domain near the trimer base (fig. S10D) (*27*). The presence of a MERS-reactive antibody in a MERS-naive donor illustrates induction of cross-reactive responses following SARS-CoV-2 infection. We were not able to reconstruct any antibodies to SARS although the SC sera had detectable titers against the spike. An overall comparison of antibody specificities between the PP and SC sera revealed antibody classes that were present in both the groups primarily targeting the S1 subunit while antibodies to the more conserved S2 subunit were enriched in the COVID donors. Collectively, these results suggest that SARS-CoV-2 infection triggers induction of cross-reactive antibodies to conserved β-CoV spike epitopes while some HCoV spike–specific antibodies may be back-boosted. This cross-boosting while associated in COVID-19 pathogenesis may also have long-lasting implications for immunity to seasonal CoVs as much of the population will be vaccinated and/or infected with SARS-CoV-2.

## MATERIALS AND METHODS

### Expression and purification of recombinant spike proteins

All spike ectodomain constructs contain a C-terminal T4 fibritin trimerization domain, an HRV3C cleavage site, an 8×His-Tag, and a Twin-strep-tag for purification. The HKU1 spike construct includes residues 1 to 1276 from isolate N5 (GenBank Q0ZME7) with the S1/S2 cleavage site modified to 752-GGSGS-756 and the residues 1067 to 1068 replaced by prolines for generating stable uncleaved spike proteins. The OC43 spike construct contains spike residues 1 to 1287 (GenBank AIL49484.1) with introduction of stabilizing prolines at sites 1079 and 1080. The SARS spike construct was generated with residues 1 to 1196 of the Tor2 strain (GenBank AAP41037.1) with stabilizing prolines at residues 968 and 969 while the MERS construct was synthesized with residues 1 to 1291 from the England1 strain (GenBank AFY13307.1) with stabilizing prolines at positions 1060 to 1061 and the S1/S2 cleavage site modified to 748-ASVG-751. For the SARS-CoV-2 spike, we synthesized a base construct (HP-GSAS) with residues 1 to 1208 from the Wuhan-Hu-1 strain (GenBank: QHD43416.1) with six stabilizing proline (HexaPro) substitutions at positions 817, 892, 899, 942, 986, and 987 and the S1/S2 furin cleavage site modified to 682-GSAS-685. We also generated three other HP-GSAS constructs each with a pair of cysteine substitutions to generate stable disulfide linkages: HP-GSAS Mut2 (S383C and D985C), HP-GSAS Mut4 (A570C and L966C), and HP-GSAS Mut7 (V705C and T883C). We used an equal ratio mixture of all four spikes for each assay (*38*, *39*).

For protein expression, FreeStyle 293-F cells (Thermo Fisher Scientific: R79007, RRID: CVCL_D603) were transected with the spike plasmid of interest and cultures were harvested at 6 days after transfection. For OC43, HKU1, SARS, and MERS, the spike proteins were purified from the supernatants on cOmplete His-Tag Purification Resin (Millipore Sigma) using a 250 mM imidazole elution buffer and buffer-exchanged to tris-NaCl buffer (25 mM tris and 500 mM NaCl, pH 7.4) before further purification with Superose 6 increase (S6i) 10/300 column (GE Healthcare Biosciences). For SARS-CoV-2 spikes, we used StrepTactin-XT 4FLOW high capacity columns (IBA Lifesciences) and elution with buffer BXT (IBA Lifesciences) before buffer exchange with buffer W (IBA Lifesciences) and S6i column purification. Protein fractions corresponding to the trimeric spike proteins were collected and concentrated. The quality of purified proteins was assessed by ns-EM.

### Human samples used in the study

For all the assays described in the paper, serum samples were used for donors 269, 1051, 1056, 1057, 1124, 1383, 1386, and 1412 while plasma samples were used for donors 1988, 1989, and 1992. The terms serum and plasma are used interchangeably in the manuscript. The studies were approved by the Institutional Review Board of Vanderbilt University Medical Center. Samples were obtained after written informed consent.

### ELISA for evaluating serum reactivity to spike proteins

To determine the EC_50_ binding titers for donor sera, we performed ELISA using 384-well plates that were coated overnight with 1 μg/ml of recombinant spike protein of interest and subsequently blocked with 5% nonfat dry milk and 2% goat serum in PBST [phosphate-buffered saline (PBS) with 0.1% Tween 20] for 1 hour at room temperature (RT). Plates were washed and 25 μl of twofold serially diluted sera starting with a fourfold dilution was added to the wells and incubated for 1 hour. The washed plates were incubated with Goat anti-human immunoglobulin G (IgG) alkaline phosphatase conjugate (Meridian Life Science, W99008A) for 1 hour and with 25 μl of phosphatase substrate solution following a final wash. The optical density values were measured at 405-nm wavelength following a 20-min incubation, and the corresponding EC_50_ values were calculated using Prism software (GraphPad) using nonlinear regression analysis. The binding assay was conducted twice independently (*n* = 2).

### HCoV-OC43 serum neutralization assay

HCT-8 cells (*Homo sapiens*, RRID: CVCL_2478) were seeded in 96-well plates at a density of 10,000 cells per well in Gibco RPMI 1640 medium with 10% fetal bovine serum (FBS; Thermo Fisher Scientific) and incubated overnight. Heat-inactivated serum was diluted in RPMI 1640 medium and incubated with OC43 virus [American Type Culture Collection (ATCC), VR-1558] for 1 hour prior. Fifty microliters of the mixture was added to each well of the 96-well plate containing the HCT-8 cells and incubated again for 1 hour before the addition of 100 μl of RPMI 1640 to each well. The plates were incubated for 4 days at 37°C, and the supernatants were harvested to perform hemagglutination inhibition assay. Fifty microliters of supernatant was mixed with 50 μl of turkey red blood cells and plated on v-bottom plates. The hemagglutination results were recorded after 30 min of incubation.

### Real-time cell analysis neutralization assay

To determine the neutralizing activity of serum/plasma against SARS and SARS-CoV-2, we used real-time cell analysis (RTCA) assay on an xCELLigence RTCA MP Analyzer (ACEA Biosciences Inc.) that measures virus-induced cytopathic effect (CPE) (*40*, *41*). Briefly, 50 μl of cell culture medium [Dulbecco’s modified Eagle’s medium (DMEM) supplemented with 2% FBS] was added to each well of a 96-well E-plate using a ViaFlo384 liquid handler (Integra Biosciences) to obtain background reading. A suspension of 18,000 Vero-E6 cells (ATCC: CRL-1586, RRID: CVCL_0574) in 50 μl of cell culture medium was seeded in each well, and the plate was placed on the analyzer. Measurements were taken automatically every 15 min, and the sensograms were visualized using RTCA software version 2.1.0 (ACEA Biosciences Inc.). Replication-competent VSV expressing SARS (VSV-SARS-CoV) or SARS-CoV-2 spike proteins (VSV-SARS-CoV-2) at 0.01 multiplicity of infection (~120 plaque-forming units per well) was mixed 1:1 with a dilution of serum/plasma or mAb in a total volume of 100 μl using DMEM supplemented with 2% FBS as a diluent and incubated for 1 hour at 37°C in 5% CO_2_. At 16 hours after seeding the cells, the virus-mAb mixtures were added in replicates to the cells in 96-well E-plates. Triplicate wells containing virus only (maximal CPE in the absence of mAb) and wells containing only Vero cells in medium (no-CPE wells) were included as controls. Plates were measured continuously (every 15 min) for 48 hours to assess virus neutralization. Normalized cellular index (CI) values at the end point (48 hours after incubation with the virus) were determined using the RTCA software version 2.1.0 (ACEA Biosciences Inc.). Results are expressed as percent neutralization in the presence of respective mAb relative to control wells with no CPE minus CI values from control wells with maximum CPE. RTCA IC_50_ values were determined by nonlinear regression analysis using Prism software.

### Serum IgG isolation and fab digestion

For IgG isolation, 1 ml of human serum diluted to 5 ml with PBS was incubated with 500 μl of washed protein G resin (GE Healthcare) overnight at 4°C. The resin was washed three times with PBS and eluted with 10 ml of 0.1 M glycine buffer at pH 2.5. The eluate was immediately neutralized with 4 ml of 1 M tris-HCl (pH 8.0) and buffer-exchanged to PBS using 100-kDa cutoff Amicon ultrafiltration units. For Fab preparation, 4 mg of concentrated polyclonal IgG samples was incubated with papain-agarose resin (Thermo Fisher Scientific) in digestion buffer (20 mM sodium phosphate, 10 mM EDTA, and 20 mM cysteine, pH 7.4) for around 22 hours in a 37°C incubator. The digest was removed from the beads and buffer-exchanged to PBS. The undigested IgGs were removed by SEC using a Superose 6 increase 10/300 column (GE Healthcare Biosciences). The purified Fabs were concentrated and assessed by SDS–polyacrylamide gel electrophoresis for purity.

### Preparation of Fab-spike complexes for ns-EMPEM

Fab-spike complexes were generated by incubating 20 μg of spike protein with 1 to 1.5 mg of purified polyclonal Fabs overnight at RT. For complexes with the SARS-CoV-2 spike, 20 μg of a mixture of spikes (GSAS-2P, GSAS-2P mut2, GSAS-2P mut4, and GSAS-2P mut7) was complexed with 5 mg of polyclonal Fabs overnight at RT. The complexes were purified on a Superose 6 increase 10/300 column using UV absorbance at 215 nm on Akta Pure system (GE Healthcare) running in tris-buffered saline (TBS) buffer. The fractions containing spike-Fab complexes were concentrated using 10-kDa cutoff Amicon ultrafiltration units and immediately used for making EM grids.

### Ns-EMPEM sample preparation and data collection

Spike-polyclonal Fab complexes diluted to approximately 20 μg/ml with TBS were directly deposited onto carbon-coated 400-mesh copper grids (made in house) and stained with 2% (w/v) uranyl-formate for 90 s immediately following sample application. Grids were imaged at 120 keV on Tecnai T12 Spirit with either a 4kx4k Tietz TemCam-F416 detector or with a 4kx4k Eagle CCD (52,000× magnification at ∼1.5 μm under focus). Micrographs were collected using Leginon and the images were transferred to Appion for processing (*42*, *43*). Particle stacks were generated in Appion with particles picked using a Difference-of-Gaussians picker (DoG-picker) (*44*). Particle stacks were then transferred to Relion for 2D classification followed by 3D classification to sort classes on the basis of different Fab specificities (*45*). Classes with similar specificities were iteratively assembled and reclassified to generate final reconstructions. A subset of 3D classes with good Fab reconstructions were auto-refined on Relion and used for making composite figures using UCSF Chimera or ChimeraX (*46*, *47*). Among the OC43- and HKU1-reactive antibodies that were detected by EMPEM 2D classes, certain specificities did not refine into 3D reconstructions as a consequence of either low particle numbers for the Fab class, orientation bias on the grid, or due to polyclonal Fab specificities targeting the same epitope. 3D refined maps were successfully generated for all OC43-reactive donors and one of two HKU1-reactive donors.

### Cryo-EMPEM sample preparation

For cryo-EMPEM studies with OC43 spike, 50 μg of the spike protein was complexed with 3 mg of purified polyclonal fab from each donor. The complex was incubated overnight at RT and purified as described above. For OC43 spike-polyclonal Fab complexes made with donor 269 and donor 1051, 3.5 μl of complex at 0.5 and 0.7 μg/ml was mixed with 0.5 μl of 0.04 mM lauryl maltose neopentyl glycol (LMNG) solution immediately before sample deposition onto 1.2/1.3 300-mesh UltraAuFoil grids (EMS). For OC43 spike-polyclonal Fab complex from donor 1412, 3.5 μl of spike-Fab complex at 0.5 μg/ml was mixed with 0.5 μl of 0.48 mM *n*-dodecyl-β-d-maltopyranoside solution before sample deposition onto 1.2/1.3 300-mesh UltraAuFoil grids (EMS). Grids were plasma-cleaned for 7 s before sample deposition using Gatan Solarus 950 Plasma system (Ar/O_2_ gas mixture). Following sample application, grids were blotted for 4 s before being plunged into liquid nitrogen–cooled liquid ethane using a Vitrobot mark IV (Thermo Fisher Scientific).

For cryo-EMPEM studies with SARS-CoV-2 spike, 40 μg of spike mixture consisting of equal ratios of HP-GSAS, HP-GSAS Mut2, HP-GSAS Mut4, and HP-GSAS Mut7 spikes was incubated overnight at RT with 10 mg of purified polyclonal Fabs (5 mg each from donors 1988 and 1989) before purifying the complexes. The complexes were mixed with LMNG immediately before sample deposition onto plasma-cleaned Quantifoil 1.2/1.3 grids (EMS) that were blotted for 3 s and plunge-frozen in liquid ethane using a Vitrobot.

### Cryo-EM data collection and processing

Micrographs were collected through Leginon software on a FEI Titan Krios operating at 300 keV mounted with a Gatan K2 direct-electron detector. The collection parameters are described in table S1. MotionCor2 was used for alignment and dose weighting of the frames and micrographs was transferred to CryoSPARC 2.9 for initial processing (*48*, *49*). CTF estimations were performed using GCTF and micrographs selected using the Curate Exposures tool in CryoSPARC based on CTF resolution estimates (cutoff 5 Å) for downstream particle picking, extraction, and iterative rounds of 2D classification and selection of intact spike trimers (*50*). The clean particle stacks were then transferred to Relion for 3D refinement and different antibody classes were sorted using the focused classification protocol described previously (*24*). The larger datasets obtained for donors 269 (~2.2 million particles post symmetry expansion) and 1051 (~2.1 million particles post symmetry expansion) resulted in reconstructions ranging from 3 to 3.5 Å resolution while the smaller dataset (~0.7 million particles after symmetry expansion) collected for 1412 resulted in reconstructions between 4 and 8 Å resolution. For SARS-CoV-2 cryo-EMPEM, five Fab-spike complexes were reconstructed at resolutions between 3.9 and 4.4 Å from a dataset of ~1.1 million particles after symmetry expansion. The data collection parameters and the processing workflow are summarized in table S1 and figs. S2, S3, S4, and S8.

### Model building and refinement

Initial model building into OC43 spike-antibody 3D maps was performed manually in Coot using PDB 6OHW as a template for the spike. Fabs are represented as poly-alanine backbone models, as their exact sequence is unknown. Iterative rounds of Rosetta relaxed refinement and manual Coot refinement were applied to generate the final models (*29*, *51*, *52*). EMRinger and MolProbity metrics were calculated following each round of Rosetta refinement to evaluate and identify the best refined models (*53*, *54*). Phenix comprehensive validation was performed on the final models. To prepare sapienic acid for modeling, PDB and crystallographic information file (CIF) ligand definition files were created using Phenix eLBOW by providing the SMILES string for PubChem CID: 5312419 (sapienic acid) (*55*). The coordinates were manually placed into their map densities in the spike and refined using Coot. Final map and model statistics are summarized in tables S2 and S4.

### Mass spectrometry

Mass spectrometry was performed as previously described to confirm the identity of sapienic acid in the apo-OC43 spike protein (*30*). Briefly, acetonitrile was used to precipitate the spike protein and extract the fatty acid followed by electrospray ionization–time-of-flight high-accuracy mass spectrometry to screen for the compound of interest in the 250 to 300 *m*/*z* (mass/charge ratio) range. We obtained a single hit at a molecular weight of 254 g/mol that matches with sapienic acid.

## Notes

View/request a protocol for this paper from *Bio-protocol*.
