# Supplementary material for: Structural mapping of antibody landscapes to human betacoronavirus spike proteins
Source: Sci Adv. 2022 May 4;8(18):eabn2911. doi: 10.1126/sciadv.abn2911 (PMC9067923; doi:10.1126/sciadv.abn2911)
Supplement: Supplementary file 1 — Figs. S1 to S10 Tables S1 to S4 [file sciadv.abn2911_sm.pdf]

Science Advances
AAAS

Supplementary Materials for

Structural mapping of antibody landscapes to human betacoronavirus spike proteins

Sandhya Bangaru, Aleksandar Antanasijevic, Nurgun Kose, Leigh M. Sewall, Abigail M. Jackson, Naveenchandra Suryadevara, Xiaoyan Zhan, Jonathan L. Torres, Jeffrey Copps, Alba Torrents de la Peña, James E. Crowe Jr., Andrew B. Ward*

*Corresponding author. Email: andrew@scripps.edu

Published 4 May 2022, *Sci. Adv.* **8**, eabn2911 (2022)
DOI: 10.1126/sciadv.abn2911

This PDF file includes:

Figs. S1 to S10
Tables S1 to S4

Figures

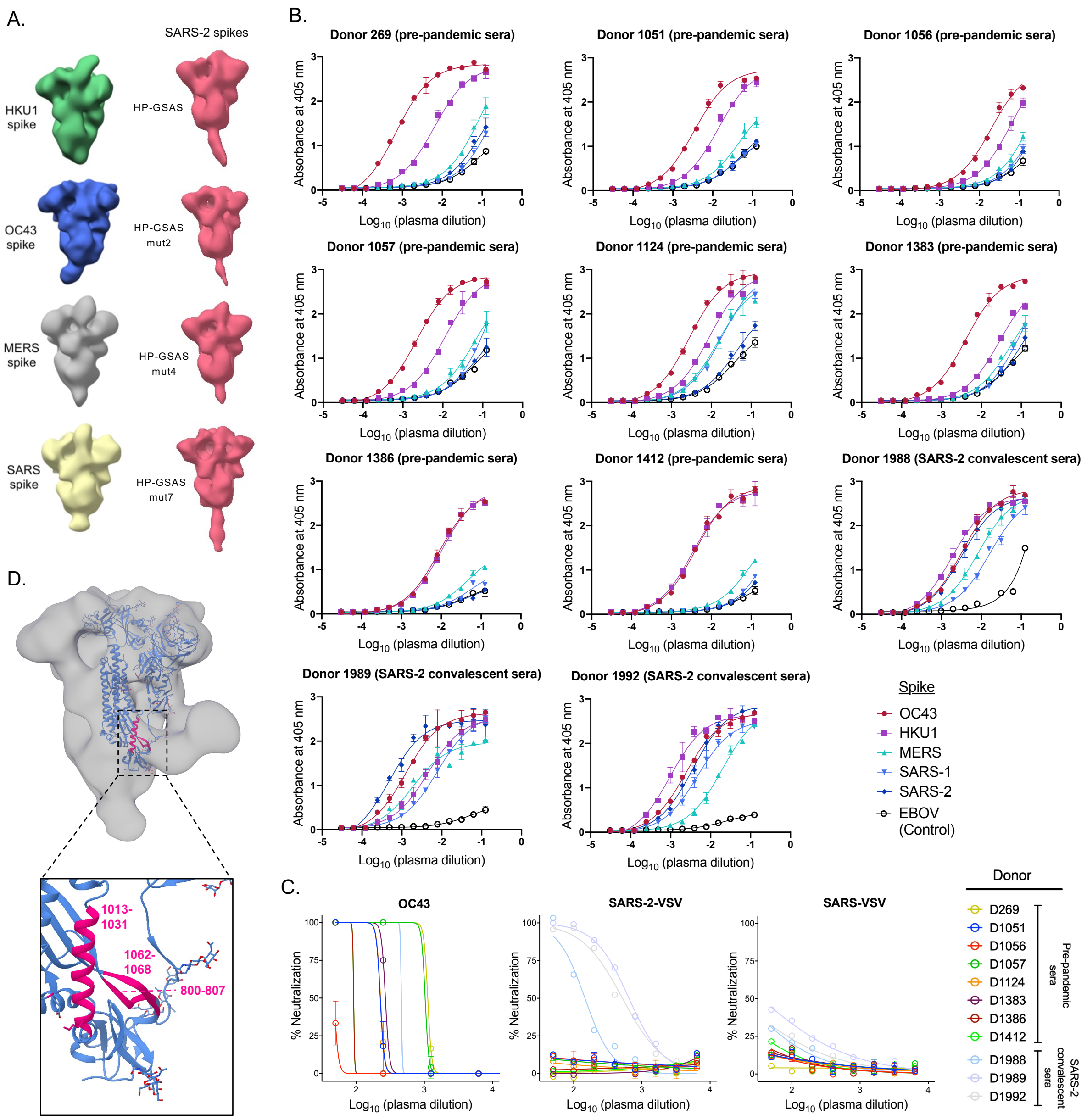

Figure S1. Serum/plasma antibody responses to β-CoV spikes (A) ns-EM reconstructions of pre-fusion stabilized spike ectodomains from OC43 (green), HKU1 (blue), MERS (grey), SARS (yellow) and SARS-2 (pink). For SARS-2, a total of four constructs are shown: a base construct (HP-GSAS) with 6 stabilizing proline substitutions and furin cleavage site modification and 3

other constructs with additional disulphide linkages, HP-GSAS Mut2 (S383C and D985C), HP-GSAS Mut4 (A570C and L966C) and HP-GSAS Mut7 (V705C and T883C). **(B)** Binding curves for 11 donor plasma/serum samples against β-CoV spikes as determined by ELISA. Ebolavirus glycoprotein (EBOV GP) was used as a control to determine baseline non-specific serum binding. The binding assay was conducted twice independently (n=2) **(C)** Serum or plasma neutralization curves for 11 donors against OC43 virus or VSV pseudotyped SARS or SARS-2 viruses. **(D)** Ns-EM 3D reconstruction (grey) of OC43 spike in complex with a S2 antibody from donor 1051. One spike protomer (blue) in the model (PDB: 6OHW) is shown docked into the EM density and a zoom-in of the antibody footprint in pink is shown in the bottom panel (*29*).

Cryo-EM analysis of HCoV-OC43 spike + polyclonal Fab from donor 269

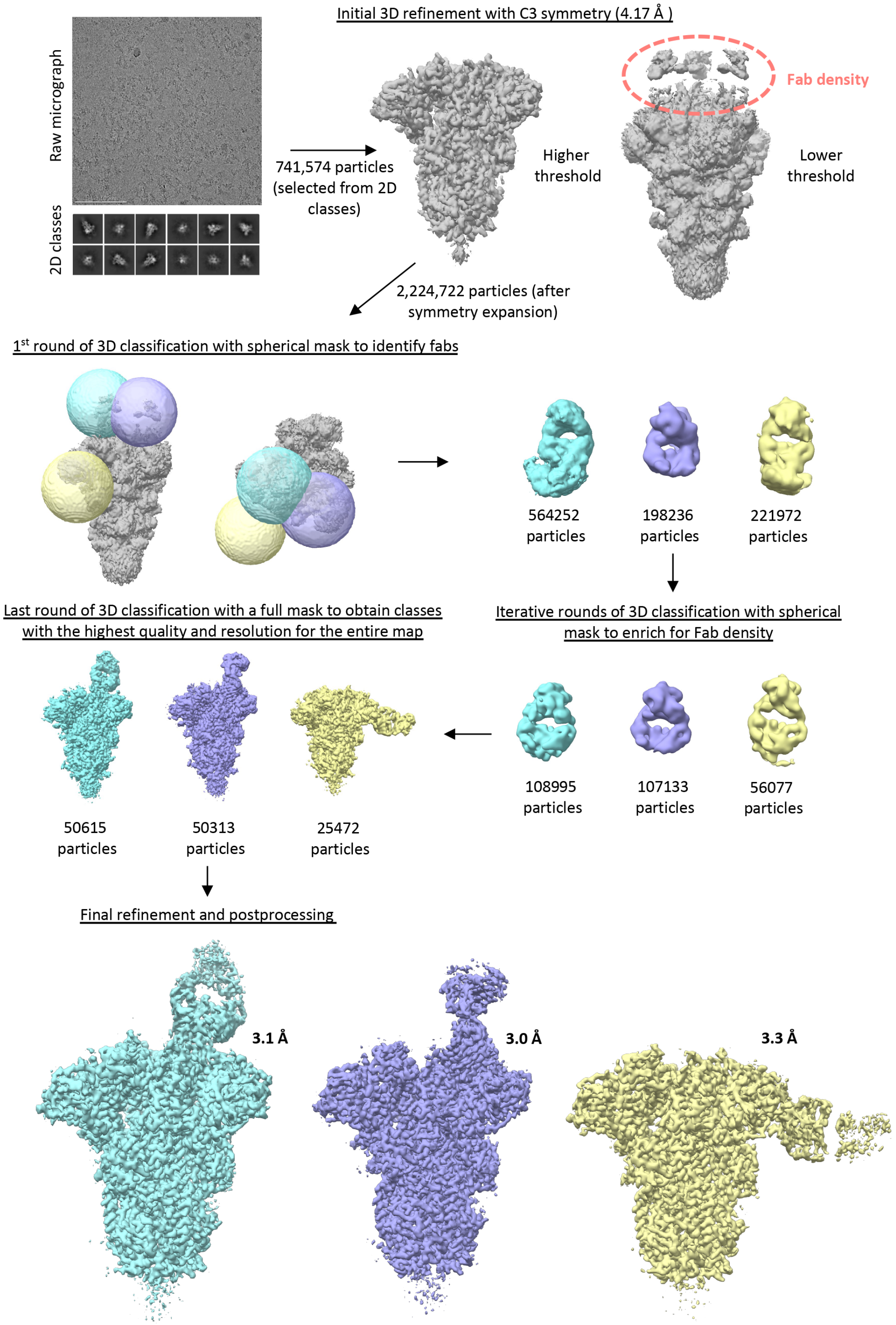

Figure S2. Schematic representation of the cryo-EMPEM processing workflow for OC43 spike complexed with polyclonal Fabs from donor 269. The focused classification approach used for generating Fab-spike reconstructions is shown in steps.

Cryo-EM analysis of HCoV-OC43 spike + polyclonal Fab from donor 1051

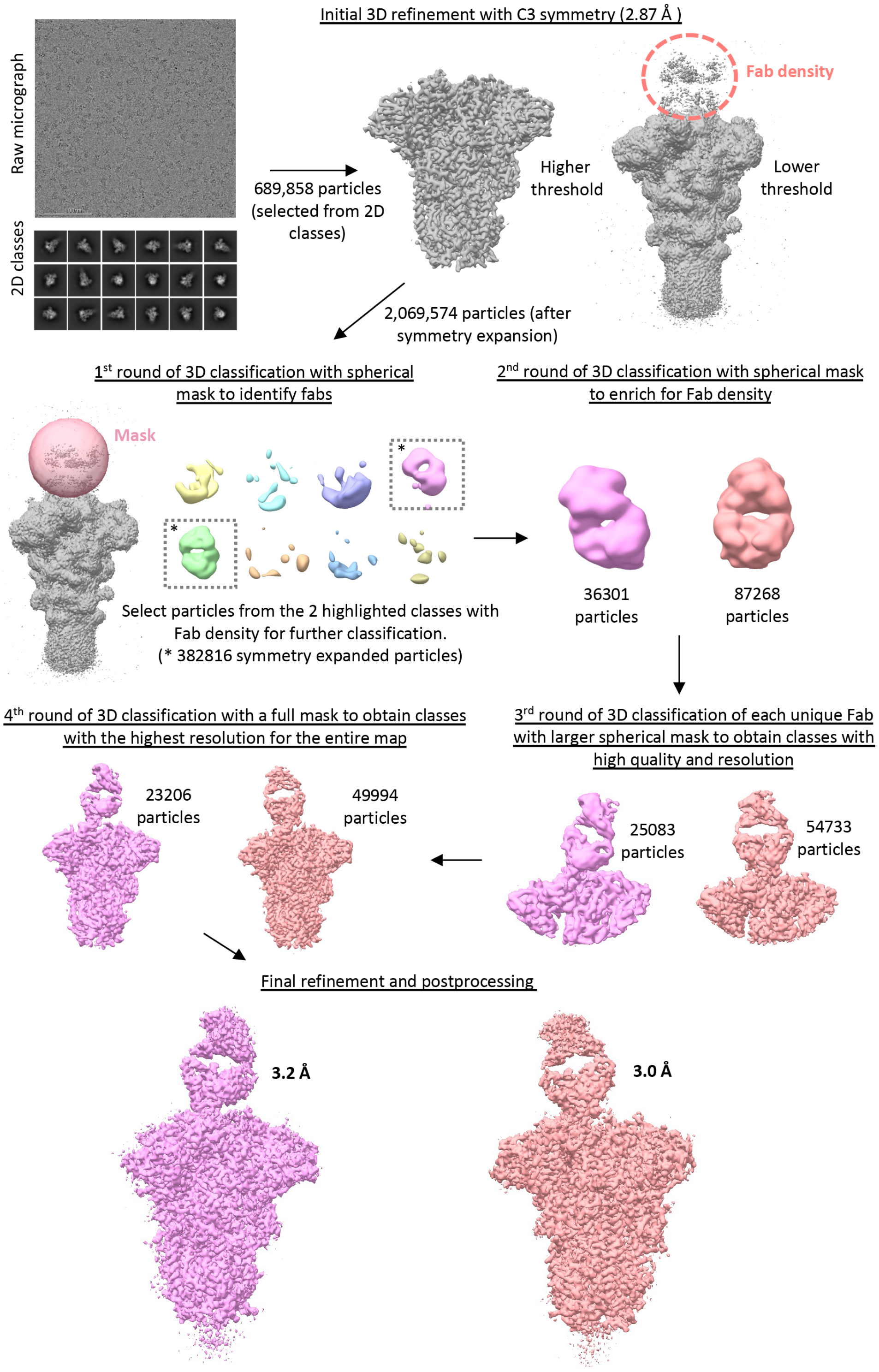

Figure S3. Schematic representation of the cryo-EMPEM processing workflow for OC43 spike complexed with polyclonal Fabs from donor 1051. The focused classification approach used for generating Fab-spike reconstructions is shown in steps.

Cryo-EM analysis of HCoV-OC43 spike + polyclonal Fab from donor 1412

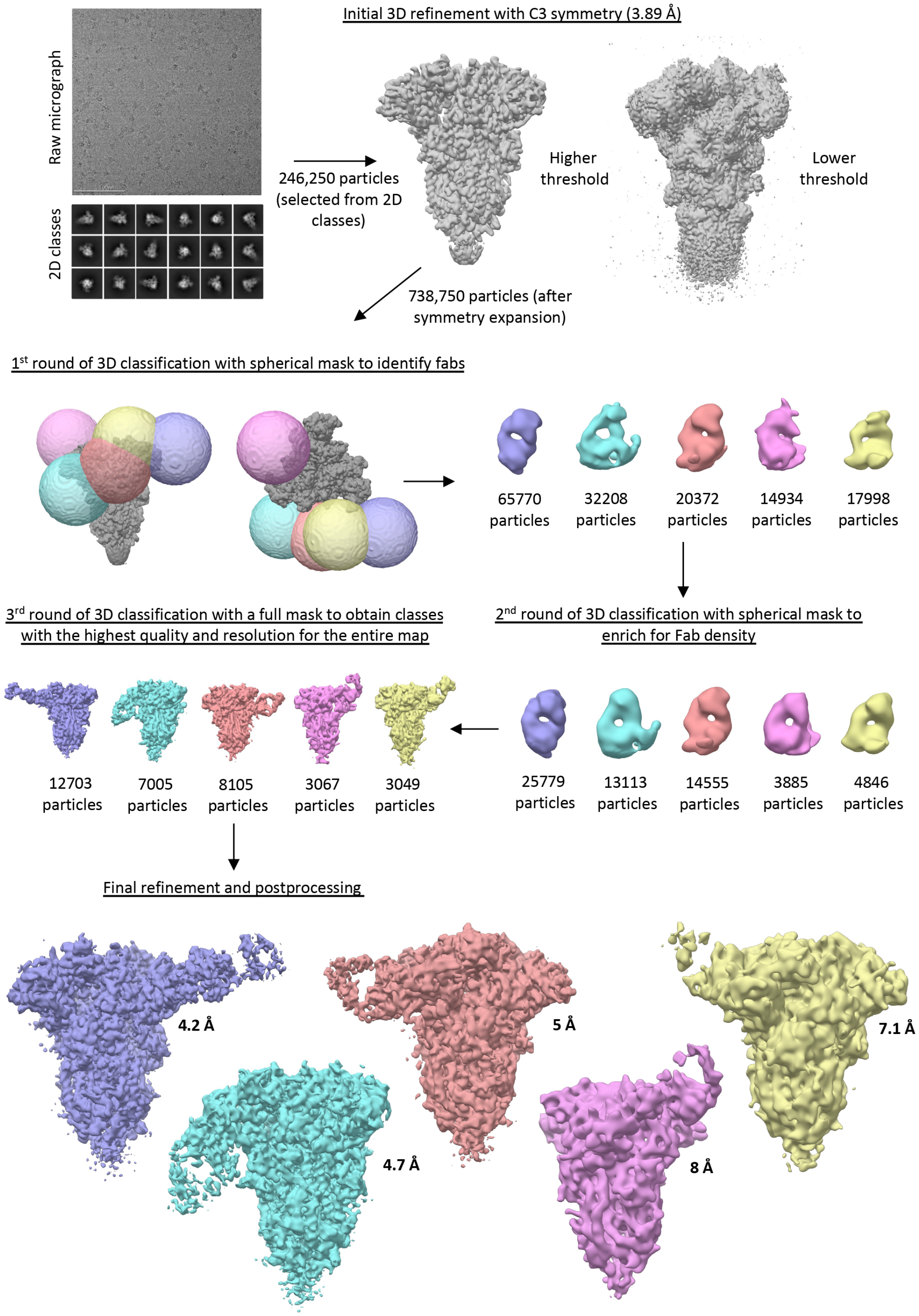

Figure S4. Schematic representation of the cryo-EMPEM processing workflow for OC43 spike complexed with polyclonal Fabs from donor 1412. The focused classification approach used for generating Fab-spike reconstructions is shown in steps.

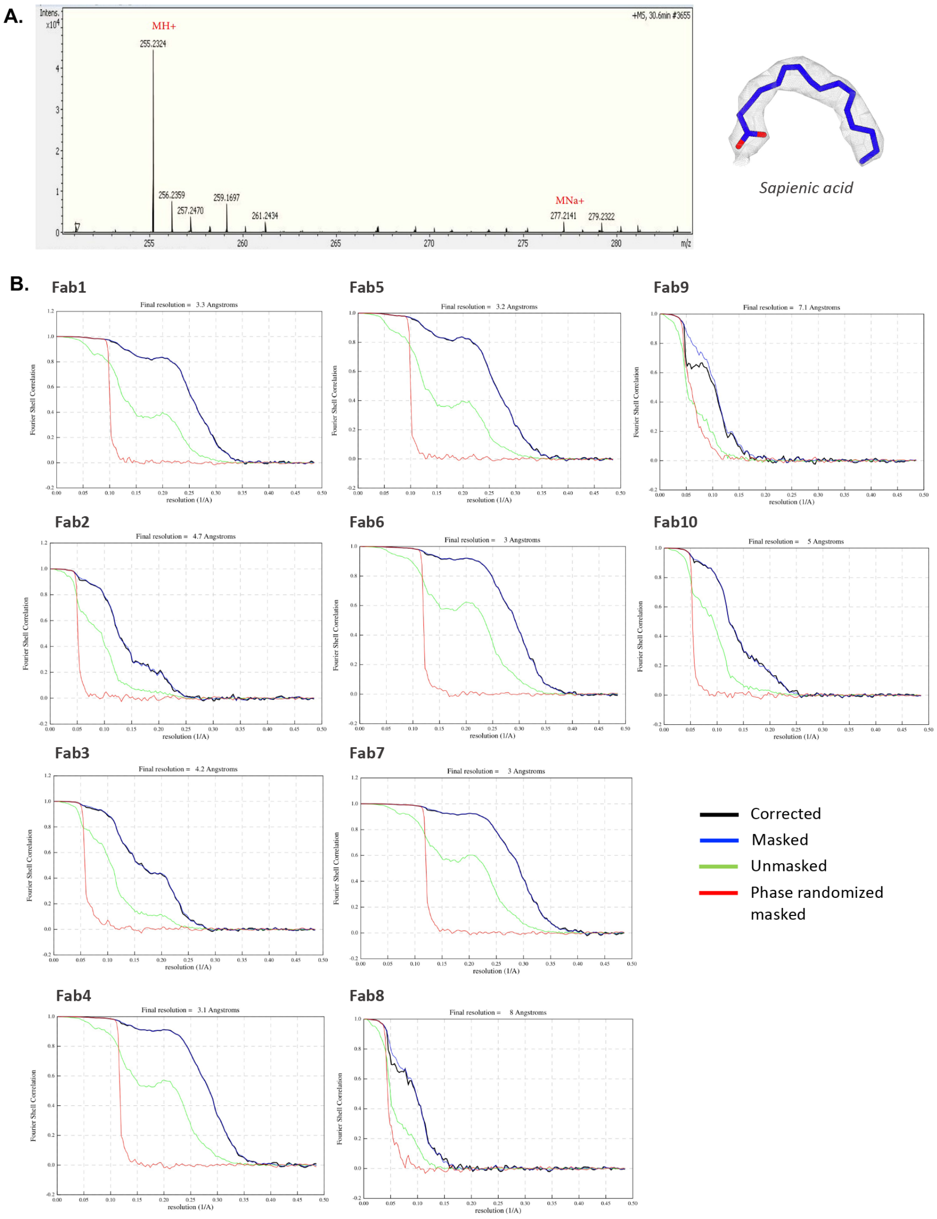

Figure S5. Cryo-EMPEM structure analysis and validation (A) Left panel shows mass spectrometry results for HCoV-OC43 spike showing a species with MW of 255 g/mol which corresponds to the protonated form of sapienic acid (MW = 254 g/mol). The right panel shows sapienic acid colored in blue surrounded by its corresponding cryo-EM map density shown as a grey mesh. Sapienic acid has a kink at position 6, originating from a cis double bond, that perfectly

recapitulates the density in our HCoV-OC43 cryo-EM maps. **(B)** FSC curves for HCoV-OC43 spike-Fab cryo-EMPEM reconstructions with C1 symmetry imposed.

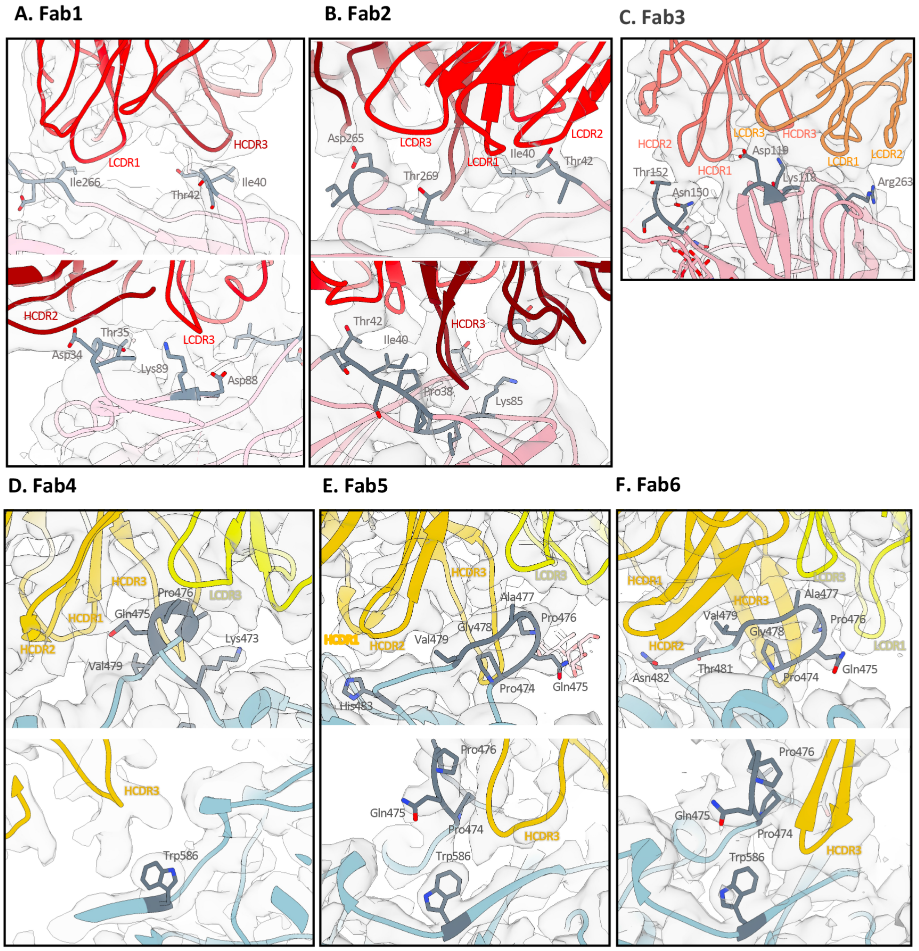

Figure S6. Epitope-paratope interactions for polyclonal Fabs 1-6 targeting the OC43 spike NTD or CTD. Ribbon representation of atomic models of OC43 spike-Fab complexes docked into their respective cryo-EM maps with close-up views of epitope-paratope interactions for (A) Fab1, (B) Fab2, (C) Fab3, (D) Fab4, (E) Fab5 and (F) Fab6. The heavy or light chains are colored dark red or light red for NTD-site 1 Fabs (Panels A and B), colored coral or orange for the NTD-site 2 Fab (Panel C), colored mustard or yellow for CTD Fabs (Panel D, E and F) and the spike is colored pink (Panels A, B and C) or blue (Panels D, E and F). The interacting residues are colored grey.

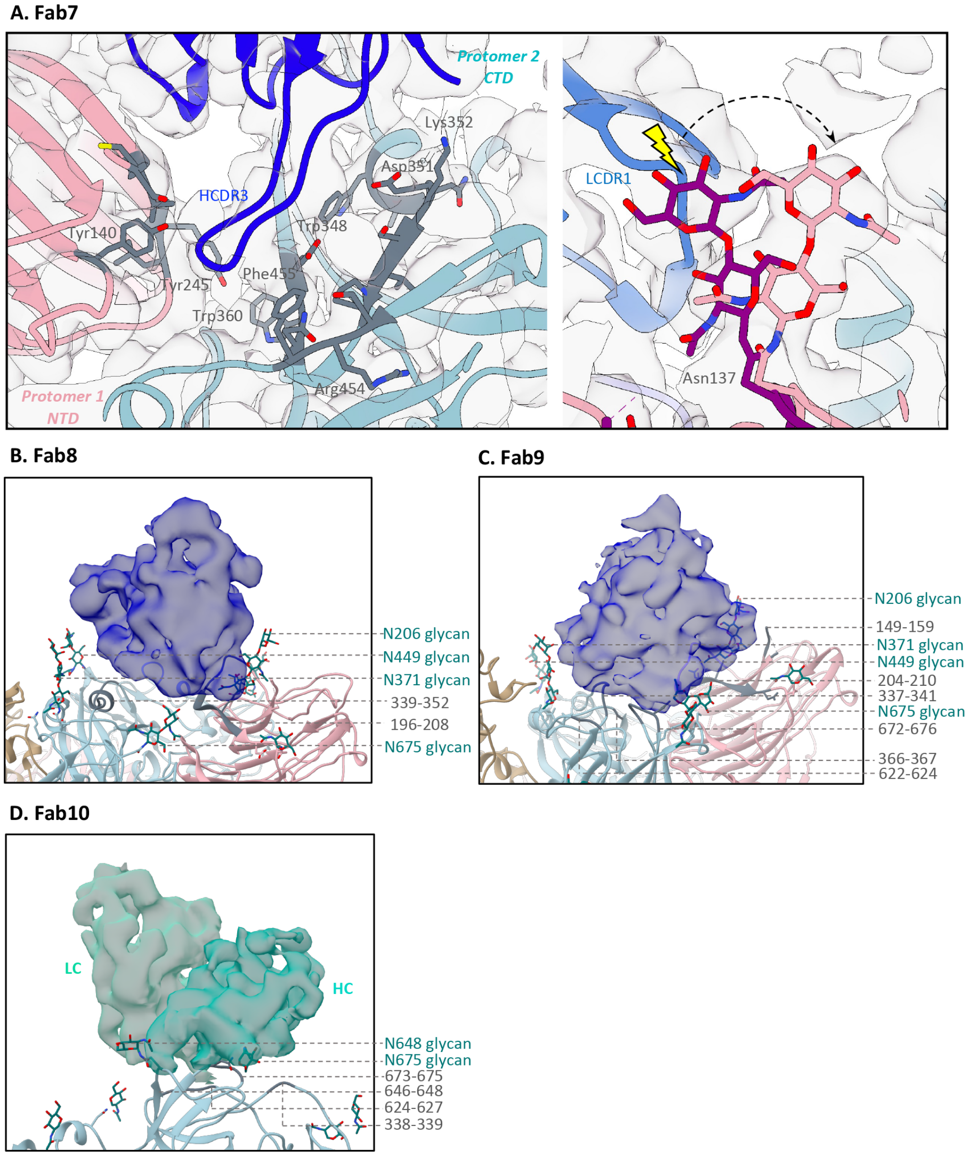

Figure S7. Epitope-paratope interactions for polyclonal Fabs 7-10 targeting the OC43 spike inter-protomeric interfaces or SD1. (A) Ribbon representation of atomic model for OC43 spike-Fab7 complex docked into the cryo-EM map with close-up views of epitope-paratope interactions. The Fab heavy and light chains are colored dark blue and light blue, respectively. The adjacent spike protomers are colored pink and blue with the interacting residues colored grey. The right panel shows displacement of N137 glycan (light pink) by LCDR3 compared to the published

structure shown in magenta (PDB 6OHW)(29). **(B and C)** Close-up views of epitope-paratope interactions for OC43 spike- **(B)** Fab8 and **(C)** Fab9 complexes displaying the cryo-EM density corresponding to the Fab in blue with ribbon representation of spike protomers in blue, pink or tan and interacting residues in grey. **(D)** Close-up view of epitope-paratope interaction for OC43 spike-Fab10 complex displaying the cryo-EM density corresponding to Fab heavy chain and light chain in shades of cyan and ribbon representation of a spike protomer in blue with interacting residues in grey. Glycans are colored teal in panels B, C and D.

Cryo-EM analysis of SARS-2 spikes + pooled polyclonal Fabs from donor 1988 and 1989

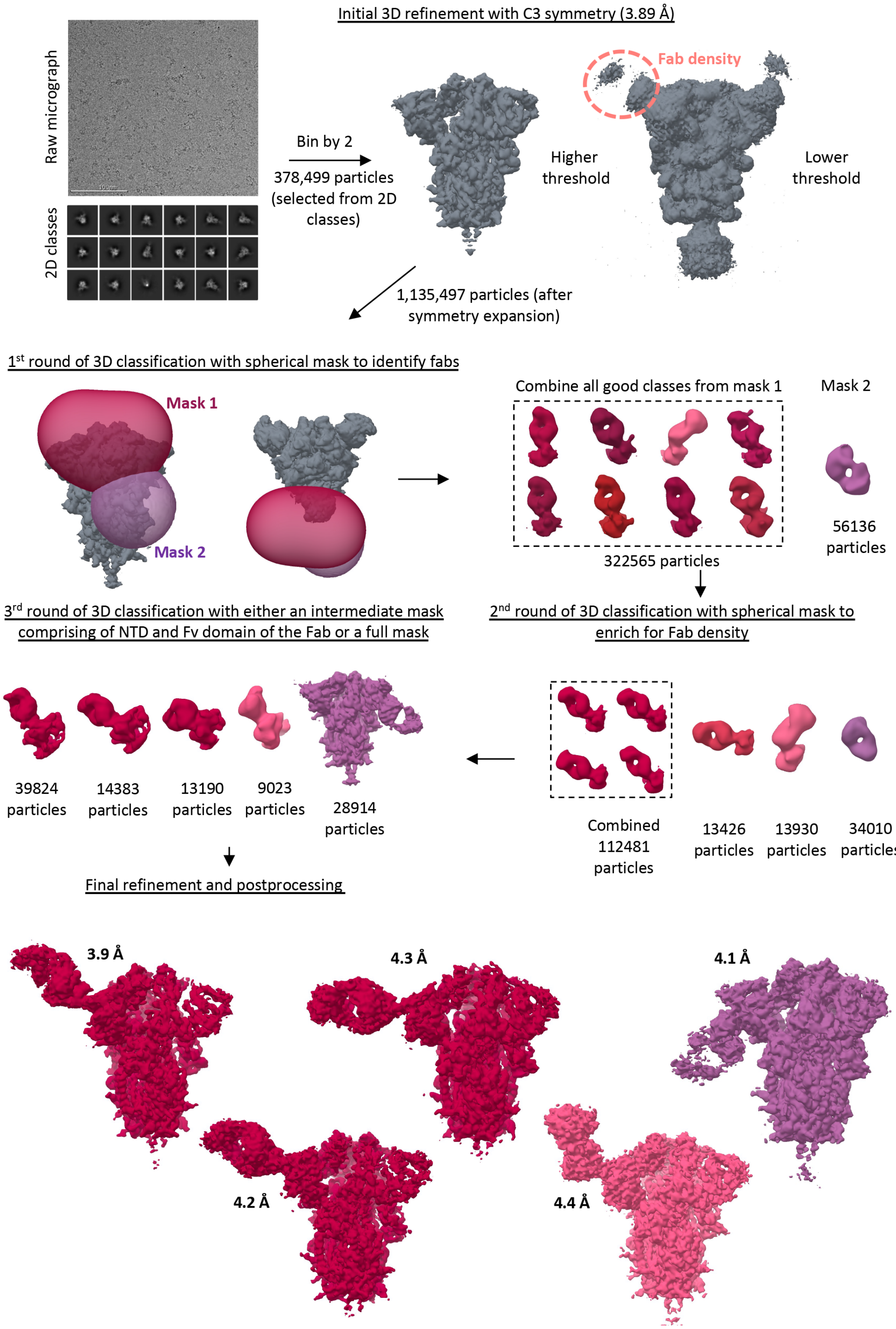

Figure S8. Schematic representation of the cryo-EMPEM processing workflow for SARS-2 spikes complexed with polyclonal Fabs from donors 1988 and 1989. The focused classification approach used for generating Fab-spike reconstructions is shown in steps.

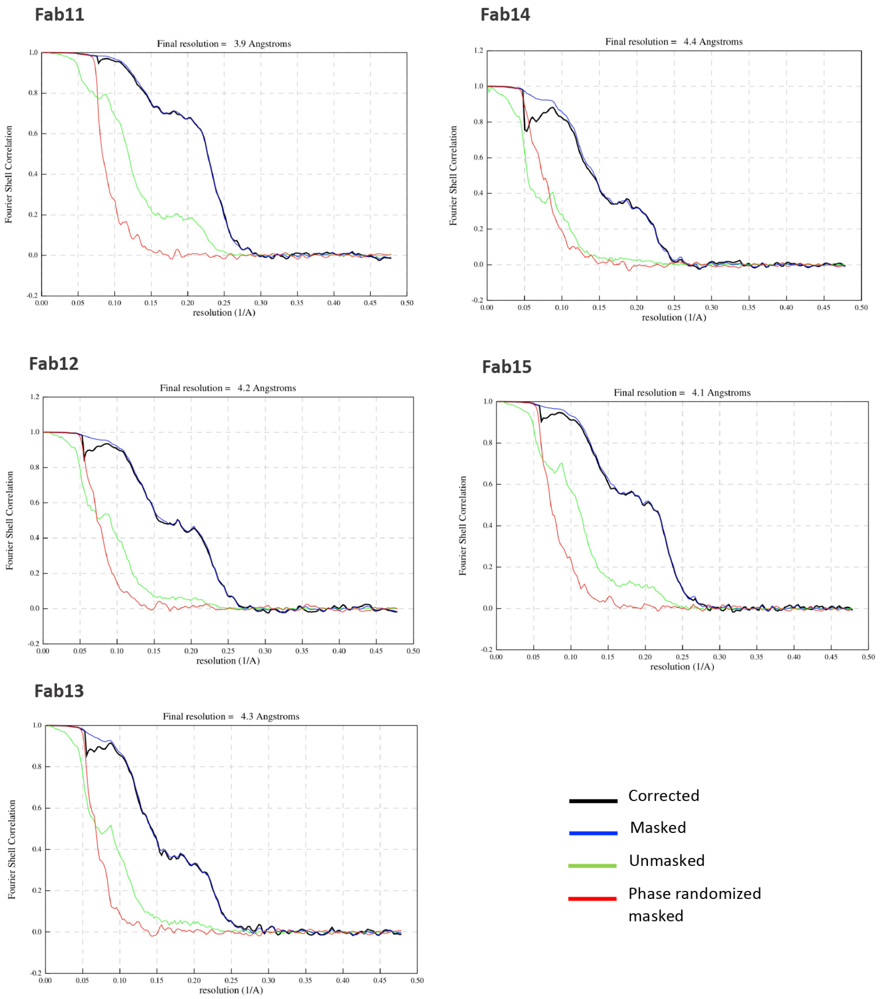

Figure S9. FSC curves for SARS-2 spike-Fab cryo-EMPEM reconstructions with C1 symmetry imposed.

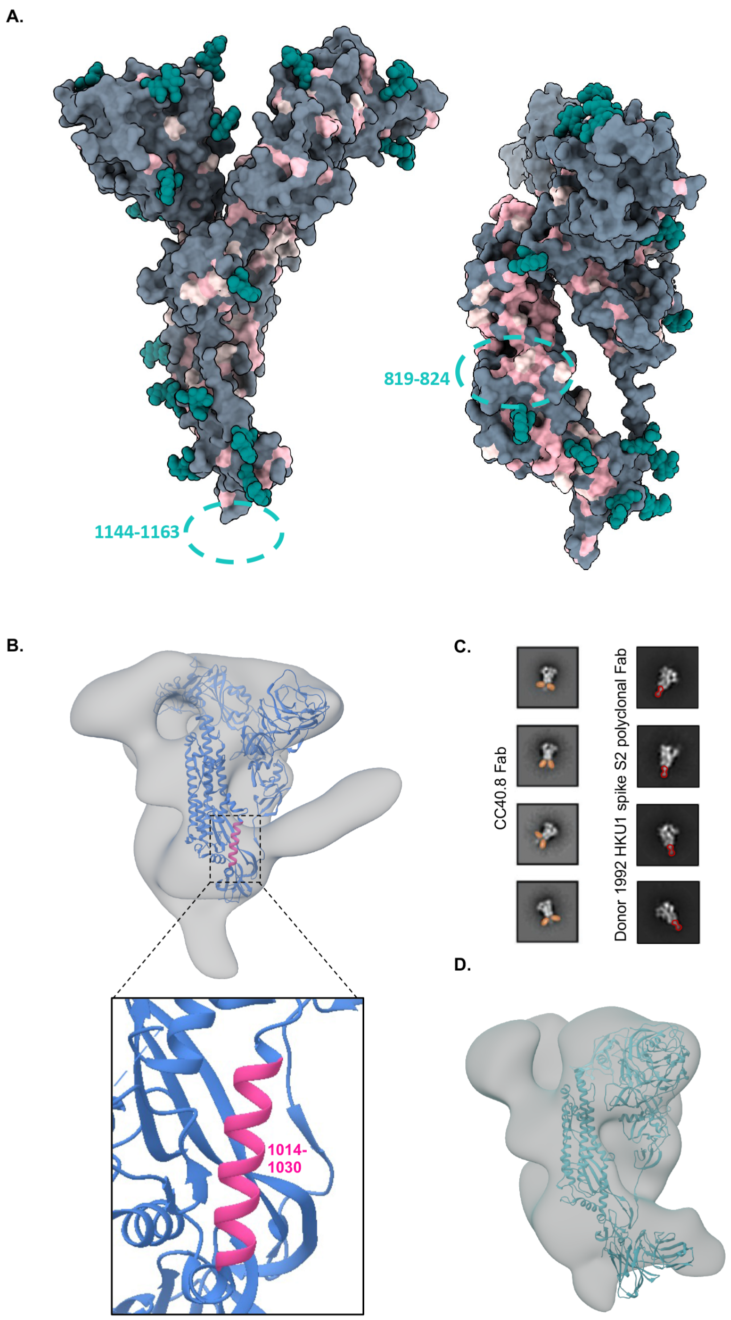

Figure S10. β-CoV cross-reactive responses in SARS-2 convalescent donors. (A) Surface representation of SARS-2 spike monomer (PDB 7JJI) colored grey and glycans colored teal. Residues on SARS-2 spike that are either identical to or have a conserved substitution in at least 3 of the 4 other β-CoVs, OC43, HKU1, SARS and MERS are colored in pink and light pink,

respectively. Conserved residues of interest from published studies (*18, 20*) are depicted on the model for reference. **(B)** ns-EM 3D reconstruction (grey) of OC43 spike in complex with a S2 antibody from donor 1989. One spike protomer (blue) in the model (PDB: 6OHW)(*29*) is shown docked into the EM density and a zoom-in of the antibody footprint in pink is shown in the bottom panel. **(C)** ns-EM 2D class averages for HKU1 spike complexed with either Fab CC40.8 (*8*) or a S2 antibody from donor 1992; the Fabs are false-colored orange and red, respectively. (D) Ns-EM 3D reconstruction (grey) of MERS spike in complex with a S2 antibody from donor 1989. One MERS spike protomer bound to G4 Fab colored in green (PDB: 5W9M) (*27*) is shown docked into the EM density for comparison.

Table S1. Cryo-EM data collection

Data collection	HCoV-OC43-269	HCoV-OC43-1051	HCoV-OC43-1412	SARS-CoV-2-1988/1989
Microscope	FEI Titan Krios	FEI Titan Krios	FEI Titan Krios	FEI Titan Krios
Voltage (kV)	300	300	300	300
Detector	Gatan K2 Summit	Gatan K2 Summit	Gatan K2 Summit	Gatan K2 Summit
Recording mode	Counting	Counting	Counting	Counting
Nominal magnification	29,000	29,000	29,000	29,000
Movie micrograph pixel size (Å)	1.03	1.03	1.03	1.045
Dose rate (e^-/[(camera pixel)*s])	5.6	5.7	5.3	7.2
Number of frames per movie micrograph	38	37	40	30
Frame exposure time (ms)	250	250	250	250
Movie micrograph exposure time (s)	9.5	9.25	10	7.5
Total dose (e^-/Å^2)	50	50	50	50
Defocus range (µm)	-0.8 to -1.5	-0.8 to -1.8	-0.3 to -1.3	-0.7 to -1.7
Number of movie micrographs	4370	2575	2321	5405

Table S2. Refinement and model building statistics for HCoV-OC43-Fab complexes

**Map**	**HCoV-OC43-Fab1**	**HCoV-OC43-Fab2**	**HCoV-OC43-Fab3**	**HCoV-OC43-Fab4**	**HCoV-OC43-Fab5**	**HCoV-OC43-Fab6**	**HCoV-OC43-Fab7**	**HCoV-OC43-Fab8**	**HCoV-OC43-Fab9**	**HCoV-OC43-Fab10**
EMDB	EMD-24968	EMD-24969	EMD-24970	EMD-24989	EMD-24990	EMD-24991	EMD-24992	EMD-24993	EMD-24994	EMD-24995
Donor	269	1412	1412	269	1051	1051	269	1412	1412	1412
Number of molecular projection images in map	25,472	7,005	12,703	50,615	25,083	54,733	50,313	3,885	4,846	14,555
Symmetry	C1	C1	C1	C1	C1	C1	C1	C1	C1	C1
Map resolution (FSC 0.143; Å)	3.3	4.7	4.2	3.1	3.2	3.0	3.0	8.0	7.1	5.0
Map sharpening B-factor (Å^2)	-64.7	-137.8	-104.5	-76.9	-63.4	-70.3	-67.9	-415.6	-242.8	-105.1
**Structure Building and Validation**										
Number of residues in deposited model										
Amino acids	3756	3739	3755	3740	3790	3769	3738			
Carbohydrates	56	55	55	56	85	62	58			
Sapienic acid	3	3	3	3	3	3	3			
MolProbity score	1.02	1.07	1.03	0.80	0.76	0.76	0.69			
Clashscore	1.85	1.93	1.62	0.54	0.78	0.84	0.50			
EMRinger score	3.62	0.83	2.06	4.5	4.32	4.9	4.74			
RMSD from ideal										
Bond length (Å)	0.018	0.019	0.020	0.022	0.022	0.023	0.021			
Bond angles (˚)	1.674	1.718	1.704	1.708	1.654	1.645	1.749			
Ramachandran plot										
Favored (%)	97.7	97.4	97.4	97.4	97.9	98	97.9			
Allowed (%)	2.3	2.6	2.6	2.6	2.1	2	2.1			
Outliers (%)	0	0	0	0	0	0	0			
Side chain rotamer outliers (%)	0.36	0	0	0.23	0.42	0.26	0.16			
PDB	7SB3	7SB4	7SB5	7SBV	7SBW	7SBX	7SBY			

Table S3. Predicted CDR lengths for HCoV-OC43 Fabs

Fab	Heavy Chain			Light Chain		
	HCDR1	HCDR2	HCDR3	LCDR1	LCDR2	LCDR3
Fab1	8	11	14	8	3	9
Fab2	8	11	14	6	2	9
Fab3	8	9	10	6	3	8
Fab4	10	11	15	4	3	9
Fab5	8	11	18	6	2	9
Fab6	8	10	19	12	3	9
Fab7	8	13	15	7	3	5

Table S4. Refinement and model building statistics for SARS-2-Fab complexes

Map	SARS-CoV-2-Fab11	SARS-CoV-2-Fab12	SARS-CoV-2-Fab13	SARS-CoV-2-Fab14	SARS-CoV-2-Fab15
EMDB	EMD-24996	EMD-24997	EMD-24998	EMD-24999	EMD-25000
Donors	1988/1989	1988/1989	1988/1989	1988/1989	1988/1989
Number of molecular projection images in map	39,824	14,383	13,190	9,023	28,914
Symmetry	C1	C1	C1	C1	C1
Map resolution (FSC 0.143; Å)	3.9	4.2	4.3	4.4	4.1
Map sharpening B-factor ($Å^2$)	-76.2	-93.1	-81.2	-90.0	-79.3
